# The Chromatin Landscape Channels DNA Double-Strand Breaks to Distinct Repair Pathways

**DOI:** 10.3389/fcell.2022.909696

**Published:** 2022-06-08

**Authors:** Zulong Chen, Jessica K. Tyler

**Affiliations:** Department of Pathology and Laboratory Medicine, Weill Cornell Medicine, New York City, NY, United States

**Keywords:** repair pathway choice, euchromatin, heterochromatin, histone modifications, transcription, RNA-DNA hybrids, DNA doube-strand breaks

## Abstract

DNA double-strand breaks (DSBs), the most deleterious DNA lesions, are primarily repaired by two pathways, namely homologous recombination (HR) and non-homologous end joining (NHEJ), the choice of which is largely dependent on cell cycle phase and the local chromatin landscape. Recent studies have revealed that post-translational modifications on histones play pivotal roles in regulating DSB repair pathways including repair pathway choice. In this review, we present our current understanding of how these DSB repair pathways are employed in various chromatin landscapes to safeguard genomic integrity. We place an emphasis on the impact of different histone post-translational modifications, characteristic of euchromatin or heterochromatin regions, on DSB repair pathway choice. We discuss the potential roles of damage-induced chromatin modifications in the maintenance of genome and epigenome integrity. Finally, we discuss how RNA transcripts from the vicinity of DSBs at actively transcribed regions also regulate DSB repair pathway choice.

## Introduction

Genome integrity is repeatedly challenged by a variety of endogenous and environmental stresses including, but not limited to, replication stress, reactive oxygen stress (ROS), ionizing radiation (IR), ultraviolet (UV) light, and various chemicals, all of which can induce DNA lesions (Tubbs and Nussenzweig, 2017). Among the various lesions, DNA double-strand breaks (DSB) are the most deleterious and can cause loss of chromosomal arms and cell death if left unrepaired and can lead to chromosomal translocations and deletions if inaccurately repaired (Elbakry and Lobrich, 2021). Defects in DSB repair lead to a variety of human diseases such as developmental defects, immunodeficiency, premature aging, neurodegenerative disorders, and cancer (Jackson and Bartek, 2009). To safeguard genome integrity, cells have evolved several DSB repair pathways, among which homologous recombination (HR) and non-homologous end joining (NHEJ) are the most predominant mechanisms (Chapman et al., 2012b; Hustedt and Durocher, 2016; Scully et al., 2019).

HR is the most faithful DSB repair mechanism and requires the presence of homologous sequences, usually the sister chromatid, as a template for DNA synthesis to accurately repair the DSB. Therefore, HR is restricted to the S and G_2_ phases of the cell cycle (Escribano-Diaz et al., 2013; Chen et al., 2018). Extensive 5′–3′ resection of the broken DNA ends is a critical step for HR repair, which is initiated by the Mre11-Rad50-Nbs1 (MRN) complex and C-terminal binding protein interacting protein (CtIP), followed by extensive resection by EXO1 or DNA2-BLM (Symington, 2014). The 3′ single-stranded DNA (ssDNA) overhangs are bound and protected by replication protein A (RPA), which is subsequently removed and replaced with the ssDNA protein RAD51 to form the ssDNA/RAD51 nucleoprotein filament. The ssDNA/RAD51 nucleofilament performs strand invasion of the homologous DNA sequence, which leads to templated DNA synthesis from the 3′ ends of the ssDNA, enabling the subsequent completion of HR (Chapman et al., 2012b).

In contrast, NHEJ demands minimal or no homology to join the DNA ends and occurs throughout the cell cycle in an error-prone manner (Escribano-Diaz et al., 2013; Hustedt and Durocher, 2016). The broken DNA ends are rapidly recognized and bound by the heterodimer of Ku70/Ku80 and form the DNA-PK complex upon binding of the DNA-dependent protein kinase catalytic subunit (DNA-PKcs) which activates a DNA damage response (DDR) signaling pathway (Blackford and Jackson, 2017). During NHEJ, the two broken DNA ends are directly joined by DNA Ligase 4 (Lig 4), through the X-ray repair cross-complementing protein 4 (XRCC4) and XRCC4-like factor (XLF) complex, with no or minimal end processing by Artemis (Lieber, 2010; Panier and Boulton, 2014).

The eukaryotic genome is packaged into chromatin within the nucleus. The fundamental unit of chromatin, the nucleosome, is composed of two copies of each core histone (H3, H4, H2A, and H2B) wrapped by 147 base pairs of DNA (Kouzarides, 2007). The unstructured core histone tails are subject to numerous different post-translational modifications (PTMs), among which phosphorylation, methylation, acetylation, and ubiquitylation have been most extensively studied (Tan et al., 2011; Rothbart and Strahl, 2014). These histone PTMs, as well as DNA methylation, help to partition the genome into distinct domains such as euchromatin and heterochromatin (which can be facultative or constitutive). Euchromatin is an open chromatin state, is associated with active transcription, and is enriched in H3K4me2/3, H3K36me3 and histone hyperacetylation (Bannister and Kouzarides, 2011; Zhang et al., 2015; Talbert and Henikoff, 2021). In contrast, heterochromatin is more highly compacted and less accessible to the transcription machinery and is enriched in repressive histone PTMs and thus transcriptionally inactive. Facultative heterochromatin is formed at regions that contain genes which are developmentally regulated and are enriched in repressive histone PTMs such as H3K9me2/3 and H3K27me2/3 (Trojer and Reinberg, 2007). Facultative heterochromatin is dispersed throughout the genome and dictates gene silencing, for example in X-chromosome inactivation, autosomal imprinted genomic loci and HOX gene clusters (Feil and Berger, 2007; Trojer and Reinberg, 2007). Constitutive heterochromatin is composed of highly repetitive sequences and is usually at gene poor regions such as centromeres, peri-centromeres and telomeres, and is characterized by repressive histone PTMs such as H3K9me3 which recruits Heterochromatin Protein 1 (HP1) (Janssen et al., 2018). Centromeric heterochromatin uniquely includes the histone H3 variant, CENP-A that epigenetically defines the location of centromeres, and is interspersed with the active histone PTMs H3K4me1/2 and H3K36me2/3, but is depleted of H3K9me3 (Bloom, 2014), which correlates with non-coding transcriptional activity at centromeric regions (Arunkumar and Melters, 2020).

The dynamics of histone PTMs on chromatin is precisely controlled by proteins that write and erase these modifications (Hyun et al., 2017). Enzymes that add and remove the chemical modifications on histones are termed “writers” and “erasers,” respectively. Proteins that recognize one specific or combination of histone PTMs are called “readers.” In response to various developmental and environmental cues, histone PTMs are actively incorporated or removed, which leads to alterations in the chromatin and gene expression. A growing body of evidence has shown that histone PTMs play crucial roles in the DNA damage response and repair (Table 1) (Chapman et al., 2012b; Dabin et al., 2016).

**TABLE 1:** Histone Marks that are involved in DSB repair choice in mammalian cells.

Histone marks	Writers	Readers	Erasers	Outcome of repair
H2AK13/15ub	RNF168 (Gatti et al., 2012; Mattiroli et al., 2012)	53BP1 (Fradet-Turcotte et al., 2013; Wilson et al., 2016), BRCA1-BARD1 (Becker et al., 2021; Krais et al., 2021), RAD18, RNF168, RNF169 (Hu et al., 2017; Kitevski-LeBlanc et al., 2017)	USP51 (Wang et al., 2016), USP44 (Mosbech et al., 2013), USP11 (Ting et al., 2019), USP3 (Sharma et al., 2014)	NHEJ, HR
H2BK120ac	SAGA (Clouaire et al., 2018)	Unknown	Unknown	HR
H2AK127/129ub	BRCA1-BARD1 (Kalb et al., 2014; Hu et al., 2021; Witus et al., 2021)	SMARCAD1 (Densham et al., 2016)	USP48 (Uckelmann et al., 2018; Densham and Morris, 2019)	HR
H3K4me2	SETD1A (Yilmaz et al., 2021)	Unknown	LSD1 (Shi et al., 2004; Yilmaz et al., 2021), JARID1 family proteins, NO66 (Højfeldt et al., 2013)	HR
H3K9me3	SUV39H1/2, SETDB1 (Alagoz et al., 2015)	HP1 (Alagoz et al., 2015), Tip60/KAT5 (Jacquet et al., 2016)	JHDM3A (Klose et al., 2006), GASC1 (Cloos et al., 2006)	HR
H3K27me3	EZH2 (Abu-Zhayia et al., 2018)	Unknown	UTX/KDM6A, UTY/KDM6C, JMJD3/KDM6B (Agger et al., 2007; De Santa et al., 2007; Walport et al., 2014)	
H3K36me2	SETMAR (Fnu et al., 2011), SETD2 (Carvalho et al., 2014; Pfister et al., 2014)	LEDGF/p75 (Daugaard et al., 2012; Pradeepa et al., 2012)	JHDM1 (Tsukada et al., 2006)	NHEJ, HR
H3K36me3	SETD2 (Carvalho et al., 2014; Pfister et al., 2014)	LEDGF/p75 (Daugaard et al., 2012; Pradeepa et al., 2012), MRG15 (Bleuyard et al., 2017), PHF1 (Musselman et al., 2012; Musselman et al., 2013)	JHDM3A (Klose et al., 2006), GASC1 (Cloos et al., 2006), JMJD2A/KDM4A (Mallette et al., 2012; Pfister et al., 2014)	HR
H3K79me2	DOT1L/KMT4 (Wakeman et al., 2012; Zhou et al., 2016)	Unknown	KDM2B/FBXL10 (Kang et al., 2018)	NHEJ
H4K12ac	Tip60/KAT5 (Umehara et al., 2010)	BRD2 (Gursoy-Yuzugullu et al., 2017)	Unknown	NHEJ
H4K16ac	Tip60/KAT5 (Tang et al., 2013)	Unknown	HDAC1, HDAC2 (Miller et al., 2010; Hsiao and Mizzen, 2013), SIRT1 (Vaquero et al., 2004), SIRT2 (Vaquero et al., 2006)	HR
H4K20me0	Unknown	BRCA1-BARD1 (Nakamura et al., 2019)	Unknown	HR
H4K20me1/2	SET8/KMT5A (Fang et al., 2002; Nishioka et al., 2002), SUV4-20H1/2 (Schotta et al., 2004)	53BP1 (Botuyan et al., 2006; Wilson et al., 2016), MBTD1 (Jacquet et al., 2016)	PHF8 (Liu et al., 2010; Qi et al., 2010), JMJD2A/KDM4A (Mallette et al., 2012)	NHEJ, HR
H4K20me3	SUV4-20H1/2 (Schotta et al., 2004; Svobodová Kovaříková et al., 2018)	53BP1 (Svobodová Kovaříková et al., 2018)	PHF2 (Stender et al., 2012), hHR23 A/B (Cao et al., 2020)	NHEJ
H4K31uf	UFM1 (Qin et al., 2019)	STK38 (Qin et al., 2020)	Unknown	HR

In a broad sense, the chromatin landscape comprises not only DNA and histone proteins but also nascent RNA transcripts that interact with the chromatin, as well as the modifications on these nucleic acids and proteins (Black and Whetstine, 2011). The spatiotemporal dynamics of these factors accurately control DNA replication, gene transcription and genome stability. DSBs occur in the context of different chromatin landscapes, which in turn activate DDR signaling pathways to remodel chromatin structure and modulate nucleosome organization such as histone variant exchange and histone post-translational modification, to facilitate DSB repair (Price and D’Andrea, 2013). Specifically, after DSB induction in mammalian cells, the chromatin structure is rapidly and transiently compacted to repress transcription, followed by the relaxation of the chromatin structure to enable access of the repair machinery, the mechanisms of which will not be covered here but have been extensively reviewed recently (Densham and Morris, 2019). Instead, here we will focus on how different chromatin structures and pre-existing and DSB-induced histone modifications orchestrate whether HR or NHEJ is used to repair a DSB, termed DSB repair pathway choice.

## Histone Modifications Orchestrate BRCA1-BARD1-and 53BP1-Mediated Double-Strand Break Repair Pathway Choice

**FIGURE 1:**
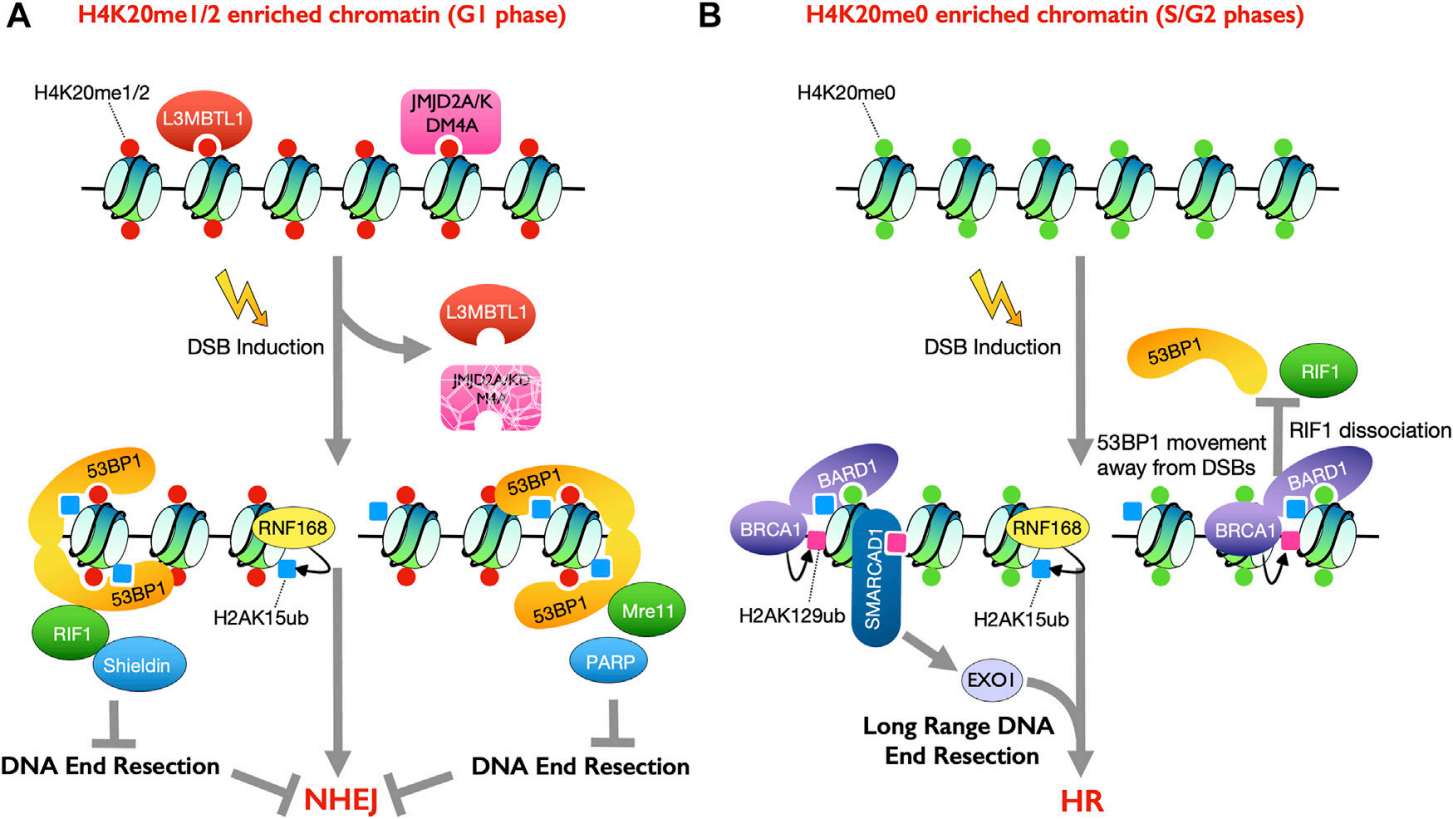
Histone modifications involved in dictating the cell cycle phase specific DSB repair pathway choice. **(A)** Histone mark H4K20me1/2 is enriched in G1 phase of the cell cycle. 53BP1 binds to nucleosomes containing both H4K20me1/2 and H2AK15ub, mediated by RNF168 upon DSB induction, to promote DSB repair by NHEJ **(B)** Unmethylated H4K20me0 is enriched in S/G2 phases of the cell cycle. BRCA1-BARD1 heterodimer recognizes H4K20me0 and H2AK15ub to facilitate HR repair (right). BRCA1 mediated H2AK129ub recruits SMARCAD1 to promote DNA end resection and thus HR (left).

Upon DSB induction, a cascade of histone and protein PTMs are induced which are critical for recruitment of DNA repair proteins. Initially, the PIKK family kinase ATM is recruited to the chromatin and activated through its interaction with NBS1 in the MRE11-RAD50-NBS1 (MRN) complex (Blackford and Jackson, 2017; Britton et al., 2013), followed by ATM phosphorylating Serine 139 on the histone variant H2A.X (γH2A.X), MDC1 and other proteins. The phosphorylated MDC1 in turn recruits an E3 ligase, RNF8, to promote K63-linked polyubiquitylation of chromatin proteins (Huen et al., 2007; Kolas et al., 2007; Mailand et al., 2007; Mattiroli and Penengo, 2021). Meanwhile, RNF8, and UBC13, an E2 ubiquitin-conjugating enzyme, mediate K63-linked polyubiquitylation on the linker histone H1, which sequentially recruits another E3 ligase RNF168 to specifically mono-ubiquitylate H2A at lysines 13 and 15 (H2AK13ub and H2AK15ub) (Mattiroli et al., 2012; Thorslund et al., 2015; Uckelmann and Sixma, 2017). Mono-ubiquitylation on H2AK13/15 in turn contributes to the recruitment of the pro-NHEJ factor p53-binding protein 1 (53BP1) (Fradet-Turcotte et al., 2013) and the pro-HR factor, the heterodimer of Breast cancer type 1 susceptibility protein (BRCA1) and BRCA1-associated RING domain protein 1 (BARD1), to DSB sites (as discussed below) (Figure 1). The mono-ubiquitylated H2AK13/15 is then further extended by RNF8 to form K63-linked ubiquitin chains to recruit downstream factors to fulfill DNA damage repair (Doil et al., 2009; Stewart et al., 2009; Mattiroli et al., 2012; Mattiroli and Penengo, 2021). In addition to 53BP1 and BARD1, H2AK15ub is also recognized by other histone readers such as RAD18, RNF169, and RNF168 itself (Hu et al., 2017; Kitevski-LeBlanc et al., 2017). Both RAD18 and RNF169 are involved in DSB repair and promote HR. Intriguingly, RNF169 and RAD18 have a much higher affinity for H2AK13/15ub, as compared to 53BP1, suggesting that RNF169 and RAD18 may be able to shift the balance between the choice of HR vs. NHEJ repair (Mattiroli and Penengo, 2021). Interestingly, DSB induced ubiquitylation or K63-linked polyubiquitylation on H2A leads to ATM-dependent transcriptional silencing. H2A deubiquitylation by the deubiquitylase USP16 is required for rapid restoration of transcription around DSB sites (Shanbhag et al., 2010). Concomitantly, DSBs within chromatin are rapidly recognized by the Ku70/80 heterodimer, which recruits and activates DNA-PKcs to promote NHEJ (Blackford and Jackson, 2017).

### H2AK15ub and H4K20me1/2 Promote 53BP1 Recruitment to Double-Strand Breaks

Whether or not DNA end resection occurs is the critical decision step in DSB repair pathway choice, as extensive DNA end resection necessitates repair by HR and blocks NHEJ (Chapman et al., 2012b). Meanwhile limited or no DNA end resection prevents HR and drives NHEJ. 53BP1 can be rapidly recruited to the vicinity of DSBs upon DSB induction, where it blocks DNA end resection (Schultz et al., 2000; Anderson et al., 2001; Rappold et al., 2001). The role of 53BP1 in blocking end resection was uncovered from a series of studies showing loss of 53BP1 rescues homologous recombination in Brca1-deficient mice and cells (Cao et al., 2009; Bouwman et al., 2010; Bunting et al., 2010). RIF1 was found, by several independent groups, to also prevent end resection at DSBs and promotes 53BP1-dependent NHEJ (Chapman et al., 2013; Di Virgilio et al., 2013; Escribano-Diaz et al., 2013; Zimmermann et al., 2013). More recently, multiple research groups identified the Shieldin complex as acting downstream of RIF1 to block DNA end resection (Dev et al., 2018; Findlay et al., 2018; Gao et al., 2018; Ghezraoui et al., 2018; Gupta et al., 2018; Mirman et al., 2018; Noordermeer et al., 2018). Once recruited to DSBs, 53BP1/RIF1/Shieldin function together to play a critical role in promoting NHEJ and blocking HR.

The recruitment of 53BP1 to DSBs, and thus NHEJ promotion, is dependent on specific histone PTMs. 53BP1 has several histone modification recognition domains (Chapman et al., 2012b). The tandem Tudor domain (TTD) in 53BP1 recognizes the mono- or di-methylated states of H4 on lysine 20 (H4K20me1/2) (Botuyan et al., 2006). Point mutation in the TTD domain of 53BP1 completely abolished its accumulation at DSBs (Huyen et al., 2004). As such 53BP1 accumulates at DSB sites marked with H4K20me2 (Pellegrino et al., 2017). However, H4K20me2 is a highly abundant histone mark that is present on the vast majority of nucleosomes in late G_2_ and G_1_ phases (Pesavento et al., 2008). Meanwhile, 53BP1 is recruited to the DNA in the vicinity of the DSBs, which are visualized in the cell as DNA repair foci upon DSB induction. As such, the DSB specific recruitment of 53BP1 must be regulated by factors in addition to H4K20me2. Indeed, structural studies showed that L3MBTL1, the human homolog of the *Drosophila melanogaster* tumor-suppressor protein l (3)mbt, and JMJD2A/KDM4A, the human histone demethylase, bound to methylated H4K20 and masked the histone interface in undamaged chromatin to prevent 53BP1 from binding to the chromatin (Huang et al., 2006; Min et al., 2007; Lee et al., 2008; Acs et al., 2011; Mallette et al., 2012). However, in response to DNA damage, both L3MBTL1 and JMJD2A/KDM4A are evicted, in a manner dependent on activation of the RNF8-RNF168 pathway upon DSB induction, which exposes H4K20me2 to enable 53BP1 accumulation at DSBs (Acs et al., 2011; Mallette et al., 2012) (Figure 1A).

Recognition of H4K20me2 is necessary, but not sufficient, for 53BP1 to be recruited to DSB sites (Zgheib et al., 2009). Accumulation of the 53BP1 orthologue in fission yeast Crb2 at DSBs relies on dual recognition of two histone marks, H4K20me2 and γH2A.X, *via* its tandem Tudor domains and C-terminal BRCT domains, respectively (Du et al., 2006; Sanders et al., 2004; Stucki et al., 2005). Damage-induced phosphorylation on the histone variant H2A.X is dispensable for 53BP1 recruitment in metazoan cells but instead the accumulation of 53BP1 on the damaged chromatin requires RNF8-RNF168-mediated histone ubiquitylation in metazoans, as mentioned above (Celeste et al., 2003; Doil et al., 2009; Stewart et al., 2009). Indeed, 53BP1 directly binds to the DSB-induced and RNF168-mediated ubiquitylation at lysine 15 on H2A (H2AK15ub) through its ubiquitylation-dependent recruitment (UDR) motif (Fradet-Turcotte et al., 2013; Wilson et al., 2016). Point mutations in the UDR motif abolish the binding of 53BP1 to H2AK15ub and attenuate 53BP1 recruitment (Fradet-Turcotte et al., 2013), demonstrating the importance of this interaction. Ultimately, the 53BP1 protein recognizes mononucleosomes containing both H4K20me2 and damage induced H2AK15ub marks, which ensures the specific recruitment of 53BP1 to damaged DNA sites in G_1_ phase cells (Figure 1A) (Panier and Boulton, 2014; Wilson et al., 2016). Cell cycle phase-dependent changes in H4K20 methylation enhance the recruitment of 53BP1 in G_1_ to promote NHEJ and reduce the recruitment of 53BP1 during S/G_2_ phases to promote HR. Using nascent chromatin capture (NCC), newly synthesized H4 histone was found to be exclusively unmethylated at lysine 20 (H4K20me0), which is a signature of post-replicative chromatin (Alabert et al., 2014; Alabert et al., 2015; Saredi et al., 2016). This unmethylated H4K20me0 exists from S phase (when most newly synthesized H4 is incorporated into the chromatin) until late G_2_/M phase, at which time the SET domain-containing protein 8 (SET8) methyltransferase catalyzes mono-methylation of H4K20 (H4K20me1) and subsequently the suppressor of variegation 4–20 homologue ½ (SUV4-20H1/2) converts the mono-methylation to di- and tri-methylation (H4K20me2/3). In this manner, most of the H4 lysine 20 is methylated by G_1_ phase (Beck et al., 2012; Jørgensen et al., 2013; Saredi et al., 2016), which promotes 53BP1 recruitment to block HR and promote NHEJ of DSBs in G_1_ phase cells (Figure 1A). This is key, because G_1_ phase cells lack sister chromatids and if HR was allowed to occur, it would result in chromosomal deletions or translocations, depending on whether the homology was on the same or a different chromosome.

### H2AK15ub and H4K20me0 Recruit BRCA1-BARD1 to Facilitate Double-Strand Break Repair by Homologous Recombination

The BRCA1-BARD1 heterodimer antagonizes 53BP1 accumulation at DSB sites in the S/G_2_ cell cycle phases (Chapman et al., 2012a; Pellegrino et al., 2017), in multiple different ways, to promote HR repair. The ankyrin repeat domain (ARD) in TONSL, a protein that forms a heterodimer with MMS22L to maintain genomic stability during replication, was identified as a reader of the unmethylated state of lysine 20 on H4 (H4K20me0) (Saredi et al., 2016). Recognition of H4K20me0 is required for the accumulation of TONSL-MMS22L at damaged replication forks and DNA lesions, which promotes RAD51 loading (Saredi et al., 2016). The ARD domains in TONSL and BARD1 are highly conserved, such that the ARD domain of BARD1 can also specifically bind to H4K20me0 (Fox et al., 2008; Saredi et al., 2016; Nakamura et al., 2019). Indeed, the presence of H4K20me0 is required for BRCA1-BARD1 recruitment to chromatin. Mutation of the predicted H4K20me0 binding motif on the ARD domain abolished BARD1 binding to nucleosomes and failed to antagonize 53BP1 accumulation at DSB sites in BARD1 deficient cells (Nakamura et al., 2019). Consistently, depletion of SET8, which in principle eliminates all methylation from H4K20, increased H4K20me0 levels and enriched BRCA1-BARD1 binding to chromatin in G_2_ and G_1_ phases to attenuate NHEJ repair (Nakamura et al., 2019).

Given that half of H4K20 is unmethylated on post-replicative chromatin in late S and G_2_ phases, how do cells ensure that the BRCA1-BARD1 complex is recruited specifically to damaged DNA to promote HR? A tandem BRCT-domain-associated ubiquitin-dependent recruitment motif (BUDR) in BARD1 was identified to recruit BRCA1 to DSB sites through its binding to H2AK15ub (Becker et al., 2021; Krais et al., 2021). Notably, as discussed above, this is one of the histone PTMs that recruits 53BP1, suggesting that 53BP1 and BRCA1-BARD1 may physically compete for binding to H2AK15ub (Figure 1). *In vitro* pull-down assays showed the interaction between GST–BARD1 (ARD–BRCT) and recombinant nucleosome variants were strongly stimulated by H2AK15ub but inhibited by methylation on H4K20 (Becker et al., 2021). On the contrary, both modifications on the histones were required for the interaction between GST–53BP1(TTD–UDR) and recombinant nucleosomes (Becker et al., 2021). Indeed, the cooperation of the ARD and BUDR domains in BARD1 to bind H4K20me0 and H2AK15ub, respectively, is required for high affinity recognition of DSB lesions by the BRCA1-BARD1 complex and for its activity in promoting HR repair on post-replicative chromatin (Becker et al., 2021; Dai et al., 2021).

### BRCA1-Mediated H2AK129ub Antagonizes 53BP1 Recruitment to Double-Strand Breaks

The BRCA1-mediated H2AK129 ubiquitylation promotes end resection via its ability to recruit SMARCAD1, an ATP dependent nucleosome remodeler that facilitates Exo1-mediated extensive DNA end resection (Chen et al., 2012; Costelloe et al., 2012; Densham et al., 2016; Eapen et al., 2012; Kalb et al., 2014; Adkins et al., 2017). Indeed, the balance between RNF168 mediated H2AK15 ubiquitylation and Brca1-mediated H2AK129 ubiquitylation is thought to determine the pathway choice between NHEJ and HR, where H2AK15ub recruits 53BP1 to promote NHEJ and H2AK129ub recruits SMARCAD1 to promote DNA end resection. On the other hand, the deubiquitylation enzyme (DUB) USP48 specifically removes the BRCA1-mediated H2Aub modifications to limit SMARCAD1 interaction to prevent over resection to limit the use of the mutagenic single-strand annealing repair pathway (Densham and Morris, 2019; Uckelmann et al., 2018) (Figure 1B). Whether USP48 is regulated in a cell cycle specific manner is unclear. Also, the DUB USP51 was shown *in vitro* to directly bind to H2A-H2B and deubiquitylate H2AK13/15ub to regulate DSB repair (Wang et al., 2016). Overexpression of USP51 suppressed IR-induced foci formation of both 53BP1 and BRCA1 (Wang et al., 2016). However, it seems that USP51 does not play a role in determining the choice between DSB repair by NHEJ or HR as it removes the PTMs on H2A that are required for both the recruitment of 53BP1 and BRCA1. Another mechanism to limit excessive resection is *via* incorporation of H2AZ at sites of damage (Xu Y. et al., 2012). H2AZ has a shorter C-terminal tail than H2A and lacks the C-terminal lysines K125/K127/K129 present on H2A, and thus H2AZ may thus be refractory to BRCA1-mediated modification and thus SMARCAD1-mediated nucleosome remodeling.

BRCA1 not only colocalizes with γH2A.X at DSB sites but also catalyzes ubiquitin conjugation to H2A.X at lysine 127, which may promote eviction of 53BP1 from γH2A.X containing chromatin (Densham et al., 2016; Hu et al., 2021; Witus et al., 2021). However, it remains unclear to what extent the ubiquitylation on H2AX by BRCA1-BARD1 contributes to DNA repair pathway choice. The ubiquitin binding protein Rap80 forms a complex with BRCA1, and this also facilitates BRCA1-BARD1 accumulation at DNA damage sites (Kim et al., 2007; Sobhian et al., 2007; Wang et al., 2007). Furthermore, the interaction between BARD1 and the nucleosome core particle inhibits K63 polyubiquitination at H2AK13ub and H2AK15ub (Hu et al., 2021). K63-linked polyubiquitylation is specifically recognized by Rap80, which recruits the Abraxas complex (ARISC) to limit end resection (Wang et al., 2007; Coleman and Greenberg, 2011; Hu et al., 2011). Consequently, because of the BARD1-mediated inhibition of K63-linked polyubiquitylation, recruitment of ARISC is compromised and HR is enhanced. As such, BRCA1 and 53BP1 antagonize each other in multiple different ways to regulate DSB repair pathway choice to promote NHEJ in G_1_ phase and HR in S/G_2_ phases.

### BRCA1’s Influence on Spaciotemporal Dynamics of 53BP1 and RIF1 Through the Cell Cycle

Histone PTMs mediate the recruitment of NHEJ- and HR- promoting factors to the chromatin flanking DSBs, which leads to changes on the spatial level of 53BP1 and BRCA1-BARD1 at DNA repair foci, as seen by microscopy. 53BP1 exists in dense foci around DSBs in G_1_ phase cells, whereas 53BP1 appears more dispersed from the DNA repair foci center in S-phase cells. Meanwhile, BRCA1, CtIP, and RPA are found at the center of DNA repair foci in S-phase cells (Chapman et al., 2012a; Kakarougkas et al., 2013). Upon experimental loss of BRCA1, 53BP1 relocalizes to the center of the S phase repair foci, resembling a G_1_ phase focus. As such, BRCA1 is responsible for the spatial positioning of 53BP1 away from DSBs in S-phase cells, and this depends on its E3 ubiquitin ligase activity (Densham et al., 2016) (Figure 1B). Recently, more details of the spaciotemporal dynamics of key DSB repair mediators during end resection at single-ended double-strand breaks (seDSB) at collapsed replication forks have been revealed by single molecule super-resolution microscopy (Whelan and Rothenberg, 2021). This study showed that 53BP1 was recruited to seDSB foci during S phase (even though seDSBs are usually repaired by HR) along with HR machinery factors such as CtIP, MRE11, BRCA1, EXO1, and DNA2. However, in contrast to the HR proteins that were retained at the damage sites for several hours, 53BP1 was rapidly removed from the seDSB foci after recruitment (Whelan and Rothenberg, 2021).

The influence of BRCA1 ligase activity on positioning 53BP1 away from DSBs in S phase cells is not the only way that BRCA1 negatively influences end protection factors. BRCA1 can also counteract RIF1 recruitment in S phase under conditions where no impact on 53BP1 is apparent (Chapman et al., 2013; Escribano-Diaz et al., 2013; Feng et al., 2013; Zimmermann et al., 2013). Specifically, BRCA1 promotes recruitment of the protein phosphatase 4°C (PP4C) to dephosphorylate 53BP1 and release RIF1 (Feng et al., 2015; Isono et al., 2017). Furthermore, BRCA1 promotes the recruitment of the protein Ub-like with PHD and RING finger domains 1 (UHRF1), which mediates K63-linked polyubiquitylation of RIF1 that results in its dissociation from 53BP1, facilitating DNA end resection and HR (Zhang et al., 2016) (Figure 1B).

## Histone Post-Translational Modifications Associated With Transcriptional Activity Promote Homologous Recombination in Euchromatin

**FIGURE 2:**
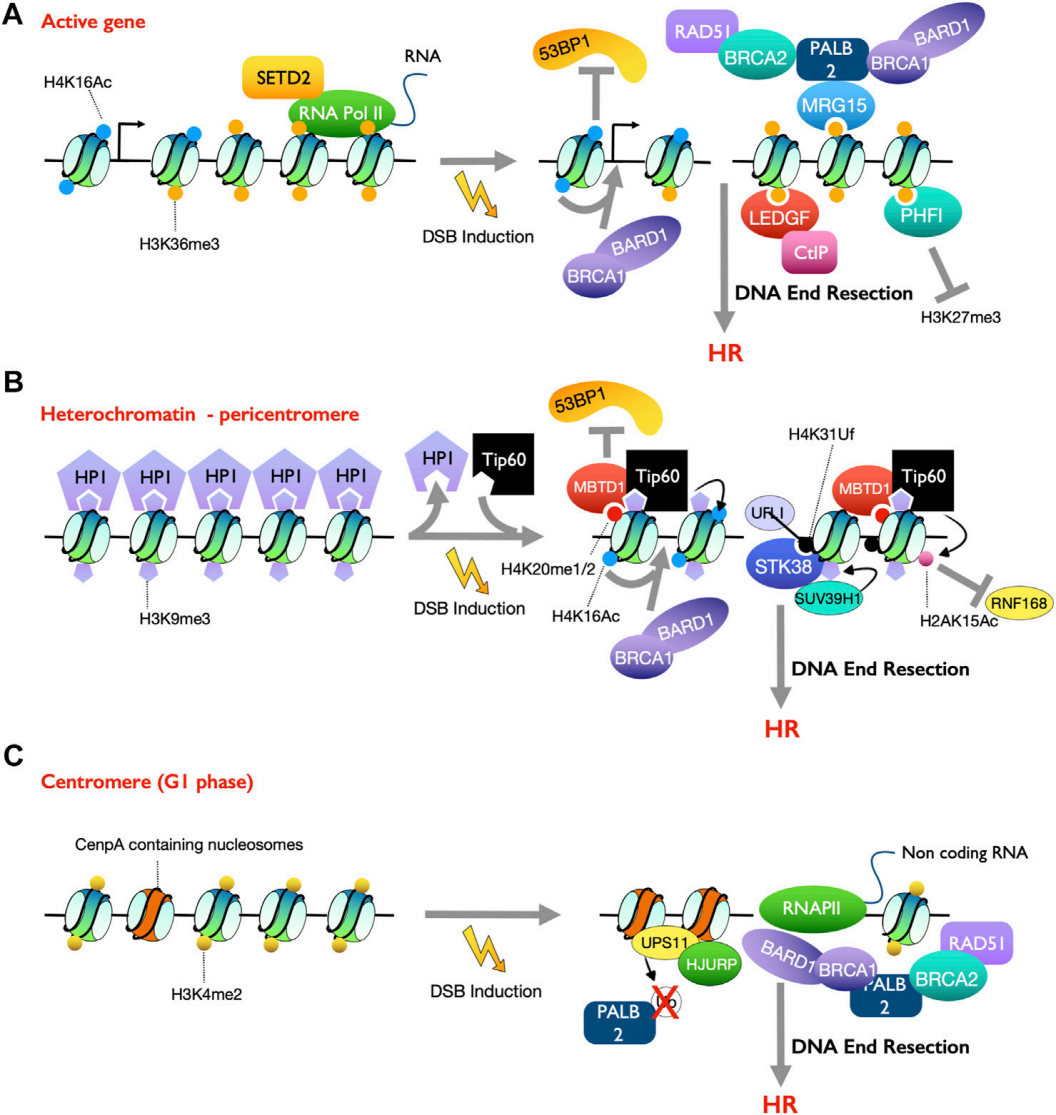
Summary of different chromatin environments that promote DSB repair by HR. **(A)** In euchromatin, transcriptionally active histone mark H3K36me3 and H4K16ac promote DSB repair by HR **(B)** At peri-centromeric heterochromatin, histone repressive mark H3K9me3 interacts with Tip60 to acetylate H4K16 to attenuate 53BP1 recruitment. MBTD1, a subunit of Tip60/NuA4 complex, competes with 53BP1 form H4K20me1/2 to promote HR repair. Upon DSB induction, UFL1 mediates ufmylation on H4K31, which recruit histone lysine methyltransferase SUV39H1 to increase H3K9me3 and thereby promote HR **(C)** At centromeres in G1 phase, H3K4me2 promotes non-coding RNA transcription and RNA-DNA hybrids formation, which recruits HR factors to DSBs. The centromere specific histone variant CENP-A promotes the recruitment of Rad51 for HR repair. The deubiquitylase USP11 stabilizes CENP-A chaperone HJURP and PALB2 to recruit RAD51 and BRCA1, respectively, for HR repair.

Mounting evidence suggests that histone PTMs that promote transcription also facilitate HR repair (Figure 2A). This is clearly the case with di- and tri-methylated lysine 36 on H3 (H3K36me2/3). The PWWP domain-containing protein lens epithelium–derived growth factor (LEDGF), the p75 splice variant of protein coding gene Psip1, is constitutively associated with chromatin via its preferential binding to H3K36me2/3 (Ge et al., 1998; Daugaard et al., 2012; Pradeepa et al., 2012). Depletion of LEDGF impairs CtIP and RAD51 recruitment to actively transcribed regions on chromatin and thus inhibits HR, which suggests that H3K36me2/3 plays a direct role in DSB repair pathway choice by promoting HR (Daugaard et al., 2012; Aymard et al., 2014). Consistently, SETD2, the main histone methyltransferase for H3K36me3, is required for HR through facilitating CtIP recruitment and end resection (Carvalho et al., 2014; Pfister et al., 2014). Further evidence for H3K36me2/3 in promoting HR comes from the fact that depletion of SETD2 impairs RAD51 recruitment and HR at DSBs within euchromatin (Aymard et al., 2014). Furthermore, overexpression of JMJD2A/KDM4A, an H3K36me2/3 demethylase, diminished HR efficiency (Pfister et al., 2014). However, neither SETD2 recruitment nor changes in H3K36me3 levels were observed at DSB sites, suggesting the pre-existing H3K36me3 at active genes promotes HR repair (Carvalho et al., 2014; Pfister et al., 2014). Conversely, DSB induction by AsiSI, an endonuclease that targets an 8-bp recognition sequence, at sites that were repaired by NHEJ, was accompanied by a significant increase in H3K36me3 and H4K20me1 (Clouaire et al., 2018), which might be due to the different approaches used to induce DSBs in these studies. Moreover, the partner and localizer of BRCA2 (PALB2) is a physical link between BRCA1 and BRCA2, thus playing a key role in DSB repair by HR (Zhang et al., 2009). Beyond being a major binding partner to BRCA2, PALB2 is associated with transcriptionally active chromatin through its interaction with MRG15 that recognizes H3K36me3 (Bleuyard et al., 2017). Another H3K36me3 reader, The PHD finger protein 1 (PHF1), is recruited to DSB sites in a manner dependent on the Ku70/Ku80 heterodimer. The recognition of H3K36me3 by PHF1 inhibits trimethylation of H3K27, which is a marker of transcriptionally silent chromatin, *in vitro* and *in vivo* (Musselman et al., 2012). Finally, binding of the PHF1 Tudor domain to H3K36me3 enhances nucleosome accessibility (Musselman et al., 2013), which may help to keep chromatin in an open state to promote HR. Therefore, H3K36me2/3, either pre-existing or induced by DSBs, plays a critical role in promoting HR repair in euchromatin through multiple different mechanisms.

In other studies, H3K36me2 was found to promote NHEJ repair at DSBs. The H3K36me2 level was markedly increased, mediated by the DNA repair protein Metnase/SETMAR containing the SET histone methylase domain, at DSB sites induced by the endonuclease I-SceI (Fnu et al., 2011). Conversely, H3K36me2 modification was reduced at DSBs induced by AsiSI (Clouaire et al., 2018). The reason for the differences in results depending on which endonuclease was induced is unclear. In fission yeast, the SETD2 homolog Set2, which mediates all three forms of H3K36 methylation, limits end resection and promotes DSB repair by NHEJ (Jha and Strahl, 2014; Pai et al., 2014). Therefore, the choice of repair pathway by NHEJ and HR appears to be carefully controlled by the methylation status of H3K36 and clearly more work remains to be done to fully understand all the nuances of H3K36 methylation in DSB repair pathway choice.

Another PTM that is linked to transcriptionally active chromatin, H4K16ac, plays an important role in tipping the balance from 53BP1 binding towards BRCA1-BARD1 binding at DSBs. Damage induced acetylation on H4K16, mediated by Tip60/KAT5, counteracts 53BP1 binding to H4K20me2 due to the disruption of the interaction between 53BP1 and H4K16 (Hsiao and Mizzen, 2013; Tang et al., 2013) (Figure 2A). Consistently, HDAC1 and HDAC2 (which deacetylate H4K16ac) rapidly accumulate at DSBs and their inactivation, either by inhibition or siRNA knockdown, reduces 53BP1 foci formation on damaged chromatin (Miller et al., 2010; Hsiao and Mizzen, 2013; Tang et al., 2013). Conversely, less H4K16ac, due to deficiency in Tip60 acetyltransferase activity, reduces BRCA1 occupancy at DSBs (Tang et al., 2013). Thus, H4K16ac promotes BRCA-BARD1 recruitment at DSBs and promotes repair by HR. Intriguingly, active transcription also promotes HR repair at DSBs that occur at regions with highly repetitive sequences such as the rDNA locus and centromeres *via* recruiting the HR machinery irrespective of the cell cycle phase (van Sluis and McStay, 2017; Yilmaz et al., 2021) (Figure 2).

### ROLES FOR DAMAGE‐INDUCED HISTONE POST-TRANSLATIONAL MODIFICATIONS IN DOUBLE‐STRAND BREAK REPAIR PATHWAY CHOICE

### Histone Post-Translational Modifications Influencing Other Histone Post-Translational Modifications in Double-Strand Break Repair Pathway Choice

It is of great interest to interrogate if DSBs can induce *de novo* PTMs on histones or removal of pre-existing histone PTMs. If so, how do these PTM changes regulate DSB repair and control repair pathway choice? H3K36me2, H3K9me3 and H4K20me3 are induced at DSBs (Fnu et al., 2011; Svobodová Kovaříková et al., 2018; Qin et al., 2020). Recently, a comprehensive mapping of histone modifications, using ChIP-seq, at DSBs induced by AsiSI was performed to gain a more complete picture of histone PTM changes during DSB repair. RAD51 ChIP-seq peaks and XRCC4 ChIP-seq peaks were defined as HR-prone sites and NHEJ-prone sites, respectively. Twenty histone PTMs and histones were interrogated in the study, among which 6 were significantly decreased (H3K79me2, H3, H3K36me2, H4K12ac, H2AZ, and H2BK120ub) and 5 were significantly increased (H4S1P, H4K20me1, macroH2A, H2BK120ac, and ubiquitin) surrounding the DSB sites (Clouaire et al., 2018). Interestingly, the switch from H2BK120 ubiquitylation (H2BK120ub) to H2BK120 acetylation (H2BK120ac) occurred at both types of DSB sites, irrespective of their being HR-prone or NHEJ-prone (Clouaire et al., 2018). The reduced H3K79me2 around DSBs may be related to the reduced H2BK120Ub because H2BK120ub stimulates DOT1L (the H3K79 methyltransferase) catalytic activity, leading to efficient methylation on H3K79 (McGinty et al., 2008; Zhou et al., 2016). Suppression of DOT1L leads to decreased recruitment of 53BP1 to DSBs (Huyen et al., 2004). Consistently, H3K79me2 is required for IR-induced 53BP1 foci formation when H4K20me2 levels are low during G_1_/G_2_ phases (Wakeman et al., 2012). Thus, the DSB induced transition of ubiquitylation to acetylation on H2BK120 and subsequent decreases in H3K79me2 may promote repair by HR if the DSB occurred at the same sites in the future. However, future studies will be required to determine to what extent these PTM changes contribute to DSB repair pathway choice.

Intriguingly, decreases in H3K36 dimethylation and H4K12/K16 acetylation after DSB induction were significant at HR-prone DSBs, whereas a significant increase in H3K36me3 and H4K20me1 was detected at NHEJ-prone sites (Clouaire et al., 2018). This is consistent with the fact that H3K36me2 facilitates NHEJ but is inconsistent with H4K16ac promoting HR, discussed above. The reduced level of H4K12ac at HR-prone sites could be related to the following mechanism by which H4K12ac indirectly recruits 53BP1: H4K12 is acetylated by Tip60 at DSBs, which recruits the bromodomain protein BRD2 to spread the acetylation state through the interaction with a second bromodomain protein ZMYND8 on the flanking chromatin (Umehara et al., 2010; Gursoy-Yuzugullu et al., 2017). BRD2 limits binding of the L3MBTL1 repressor to expose H4K20me1/2 and thus promotes 53BP1 recruitment (Gursoy-Yuzugullu et al., 2017). In line with this report, ZMYND8 was shown to be recruited to damaged chromatin by Tip60 mediated acetylation on H4 in transcriptionally active chromatin (Gong et al., 2015). However, ZMYND8 has also been reported to promote DSB repair by HR at breaks induced by AsiSI (Gong et al., 2015; Gong et al., 2017), which suggests that there is more to learn before we understand the intricate dynamics between NHEJ and HR repair. The increase in H3K36me3 following DSB induction at the NHEJ-prone sites is consistent with the reports of H3K363 promoting NHEJ (although as discussed above, some reports propose that H3K36me3 promotes HR), while induction of H4K20me1 around NHEJ-prone DSBs is consistent with elevated 53BP1 recruitment and NHEJ.

### The Role of Histone Variant macroH2A1 in Double-Strand Break Repair Pathway Choice

The histone H2A variant macroH2A1 has two alternative splicing isoforms, macroH2A1.1 and macroH2A1.2, with distinct biological functions (Kim et al., 2018). MacroH2A1.1 was found to accumulate at DSBs dependent on PARP1 (Timinszky et al., 2009; Xu C. et al., 2012). In agreement, incorporation of macroH2A1 into chromatin was significantly increased at AsiSI induced DSBs (Clouaire et al., 2018). Interestingly, either overexpression or depletion of macroH2A1.1 leads to impaired NHEJ repair due to a reduction in the recruitment of the Ku70/Ku80 heterodimer or 53BP1, respectively (Timinszky et al., 2009; Xu C. et al., 2012). MacroH2A1.1 was shown to cooperate with PARP-1 to stimulate H2BK120 acetylation, which is consistent with the ubiquitylation to acetylation transition on H2BK120 after DSB repair (Chen et al., 2014; Clouaire et al., 2018). Recently, macroH2A1.1 was shown to promote DSB repair by micro homology mediated end joining (MMEJ), also termed alternative end joining, in mice (Sebastian et al., 2020). Likewise, the macroH2A1.2 variant was also recruited to DSBs, mediated by ATM, and recruits BRCA1 to facilitate HR repair (Khurana et al., 2014). Taken together, both macroH2A1 variants play important roles in regulating DSB repair pathway choice. In this study (Clouaire et al., 2018), whether macroH2A1, macroH2A1.1 or macroH2A1.2, or both were increased at the vicinity of DSB sites is unclear since a macroH2A1 antibody was used to conduct the chromatin immunoprecipitation assay. In the future, it is worth differentiating the changes of levels of macroH2A1.1 and macroH2A1.2 at DSBs, as it appears likely to control the balance of DSB repair pathway choice given they promote NHEJ and HR, respectively.

### Double-Strand Break Induced Novel Histone Post-Translational Modifications Regulate Repair

Novel histone PTMs have also been discovered at DSB sites that regulate repair. UFM1 specific ligase 1 (UFL1), an ufmylation E3 ligase, was identified to be recruited to DSB sites by the MRN complex (Qin et al., 2019). The recruitment of UFL1 and ufmylation on MRE11 is important for ATM activation, while serine 462 on UFL1 is phosphorylated by ATM to enhance its E3 ligase activity (Qin et al., 2019; Wang et al., 2019). UFL1 also monoufmylates histone H4 at lysine 31 (H4K31uf), which is recognized by the serine/threonine kinase 38 (STK38) to recruit SUV39H1, through the HP1/KAP-1 complex, leading to an increase in the heterochromatin mark H3K9me3 (Qin et al., 2020) (Figure 2B). Moreover, the zinc-finger domain containing proteins, ZMYM2 and ZMYM3, were identified as antagonizers of 53BP1 recruitment to facilitate HR factor recruitment at DSBs (Lee et al., 2022). Recruitment of ZMYM2 to DSB sites requires the SUMO E3 ligase PIAS4 and SUMO binding activity of ZMYM2 (Lee et al., 2022), suggesting that either sumoylated HR proteins or histones around the DSBs may mediate DSB repair pathway choice. Indeed, H2A.X is sumoylated by PIAS4 at DSBs (Chen et al., 2013). Also, sumoylation was identified, *in vivo* and *in vitro*, on histone H4 but it is unknown if it is stimulated by DNA damage and how it regulates repair (Shiio and Eisenman, 2003). It is likely that other new types of histone PMTs will be found that regulate DSB repair pathway choice in the future.

## Double-Strand Break Repair Pathway Choice in Heterochromatin Is Coordinated by Chromatin Decompaction and Histone PTMs

### H3K9me3 Promotes Homologous Recombination Repair While H3K27me3 Promotes Non-Homologous End Joining Within Heterochromatin

H3K9me3 promotes HR repair and reduces NHEJ, seemingly by a combination of mechanisms. H3K9me3 is enriched at heterochromatin regions, but also increases at DNA damage sites to form transient repressive chromatin in euchromatin (Ayrapetov et al., 2014; Tsouroula et al., 2016). Several H3K9me3 writer proteins, SUV39H1/2, and SETDB1, and reader proteins, HP1, and TIP60, were shown to promote HR repair (Alagoz et al., 2015; Jacquet et al., 2016). Furthermore, the interaction between H3K9me3 and Tip60 at DSB sites, together with phosphorylation of Tip60, activates the acetylase activity of Tip60, which in turn acetylates histone H4K16 to attenuate 53BP1 binding to chromatin (Sun et al., 2005; Sun et al., 2009) (as discussed above). MBTD1, a stable subunit of the TIP60/NuA4 complex that acetylates H4K16, competes with 53BP1 for binding to H4K20me1/2. Finally, TIP60 acetylates H2AK15 and blocks its ubiquitylation by RNF168, which leads to blocking 53BP1 binding to the chromatin and promoting HR (Tang et al., 2013; Jacquet et al., 2016) (Figure 2B). In this manner, upon DSB induction, H3K9me3 promotes H4K16ac and H2AK15ac which reduce 53BP1 binding to limit NHEJ in constitutive heterochromatin.

In facultative heterochromatin, the repressive histone mark H3K27me3 is increased after DSB induction (Abu-Zhayia et al., 2018). This occurs by the DSB induced recruitment of the methyltransferase enhancer of zeste 2 (EZH2) by chromodomain Y-like (CDYL1) to catalyze methylation on H3K27 (Abu-Zhayia et al., 2018). Conversely, DSBs induced by CRISPR-Cas9 guided cutting in H3K27me3 enriched chromatin undergo MMEJ while inhibition of EZH2 leads to NHEJ repair (Sallmyr and Tomkinson, 2018; Schep et al., 2021). Moreover, another heterochromatin enriched histone PTM H4K20me3 is induced by irradiation in a manner dependent on SUV39H1/H2, which may promote 53BP1 recruitment (Svobodová Kovaříková et al., 2018).

There also exist mechanisms to limit HR within heterochromatin, including mechanisms to move the DSBs to the edge of the heterochromatin domain. Heterochromatin is enriched for highly repetitive sequences, where HR repair may lead to mutagenic recombination (Janssen et al., 2018). *Drosophila* lysine demethylase 4a (dKDM4a) is recruited to DSBs in pericentromeric heterochromatin, but not in euchromatin, and promotes the demethylation of heterochromatic histone marks, H3K9me3 and H3K56me3, to limit HR in heterochromatin domains (Janssen et al., 2019). This may promote the chromatin decompaction that is required for efficient DSB repair at heterochromatic regions. In *Drosophila,* DSBs in heterochromatin are relocated in the nucleus, driven by F-actin and myosins, to the periphery of the heterochromatin domains and then move to the nuclear pore for repair (Chiolo et al., 2011; Ryu et al., 2015; Caridi et al., 2018). In mammalian cells, relocation of heterochromatic DSBs to the periphery of heterochromatin was also observed, indicating that this movement of heterochromatic DSBs is conserved (Jakob et al., 2011; Tsouroula et al., 2016). The purpose for this relocation of DSBs within repeated sequence to the periphery of heterochromatin enables RAD51 association, because RAD51 is recruited only after relocation of the DSB out of the heterochromatin domain to promote HR repair. The mechanism whereby RAD51 is prevented from binding to DSBs within repeated sequences while they are located within the heterochromatin domain is mediated by the Smc5/6 complex, presumably to prevent inaccurate recombination which will occur if the broken DNA repeat is repaired within the repeat-rich environment of heterochromatin domains (Chiolo et al., 2011).

### H3K4me2 and CENP-A Facilitate Double-Strand Break Repair by Homologous Recombination at Centromeres in G_1_ Phase Cells

Intriguingly, DSB repair at centromeres and peri-centromeres, though both defined as constitutive heterochromatin, is strikingly different. At pericentromeric heterochromatin, DSBs induced by CRISPR-Cas9 in G_1_ are not relocated out of the heterochromatin domain and recruit NHEJ proteins, while in G_2_ they relocate to the periphery of heterochromatin for HR repair (Tsouroula et al., 2016). However, DSBs at centromeric regions recruit both NHEJ and HR factors throughout the cell cycle (Tsouroula et al., 2016). It is possible that these differences are related to the fact that peri-centromeres are enriched for the repressive PTM H3K9me3 and its HP1 reader, while centromeres include CENP-A and active chromatin PTMs such H3K4me2, H3K36 methylation and H3 acetylation, but are depleted of H3K9me3 (Chan and Wong, 2012; Bloom, 2014).

HR repair requires the presence of sister chromatids and is usually suppressed in the G_1_ phase of the cell cycle. Interestingly, the HR machinery was recently reported to also be recruited to DSBs at centromeres in G_1_ phase even in the absence of sister chromatids (Yilmaz et al., 2021). The active histone mark H3K4me2 at centromeres promotes non-coding RNA transcription. This was shown by the depletion of the histone methyltransferase SETD1A or tethering of the H3K4me2 demethylase LSD1 to centromeres leading to a substantial decrease in transcription of centromeric non-coding RNAs (Yilmaz et al., 2021). Interestingly, DSB breaks at centromeres, but not peri-centromeres, in G_1_ phase increased centromeric transcription and concomitantly the H3K4me2 level, which in turn facilitated RNA-DNA hybrid formation (Aguilera and Gómez-González, 2017; Yilmaz et al., 2021). The RNA-DNA hybrids are required for the recruitment of HR components including BRCA1, RPA, and RAD51 to promote end resection and HR repair in G_1_. In addition to promoting transcription at centromeres, H3K4me2 was also shown to be required for HJURP (the histone H3 variant CENP-A chaperone)-mediated CENP-A assembly onto an epigenetically engineered human kinetochore (Bergmann et al., 2011). Depletion of the centromeric histone H3 variant CENP-A, its chaperone HJURP or the cofactor MIS18 leads to a significant decrease in recruitment of RAD51 at DSBs in centromeres (Yilmaz et al., 2021). This suggests that CENPA is important for HR, *via* RAD51 recruitment, to DSBs within the centromere in G_1_ cells. Indeed, dCas9-mediated tethering of CENP-A or HJURP at pericentromeric DSBs increased the recruitment of RAD51 in G_1_, suggesting that CENP-A and HJURP are sufficient to recruit RAD51 (Yilmaz et al., 2021). Ubiquitylation on PALB2 in G_1_ phase suppresses its interaction with BRCA1 and thus RAD51 recruitment, which is counteracted by the deubiquitylase USP11 (Orthwein et al., 2015). USP11 is rapidly turned over in G_1_, especially upon DSB induction (Orthwein et al., 2015). Interestingly, USP11 was found to be specifically recruited to centromeric, but not peri-centromeric, DSBs in G_1_ and interacts with both CENP-A and HJURP to promote RAD51 recruitment (Yilmaz et al., 2021). Furthermore, USP11 deubiquitylates HJURP which is normally ubiquitylated to facilitate CENP-A incorporation at centromeric chromatin (Yilmaz et al., 2021), which thereby reinforces HR repair at centromeres in G_1_. Indeed, depletion of USP11 leads to impairment in RAD51 recruitment and HR repair at centromeres in G_1_ (Yilmaz et al., 2021). Mechanistically, the presence of H3K4me2 and HJURP-CENP-A at centromeres promotes HR in G_1_ phase (Yilmaz et al., 2021). In summary, there are complex mechanisms to regulate DSB pathway choice in centromeric and peri-centromeric heterochromatin (Figures 2B,C).

## Double-Strand Break Induced RNA Transcripts Play Pivotal Roles in Double-Strand Break Repair Pathway Choice

### Non-Coding RNAs: diRNAs/DDRNAs and dilncRNAs in Double-Strand Break Repair Pathway Choice

**FIGURE 3:**
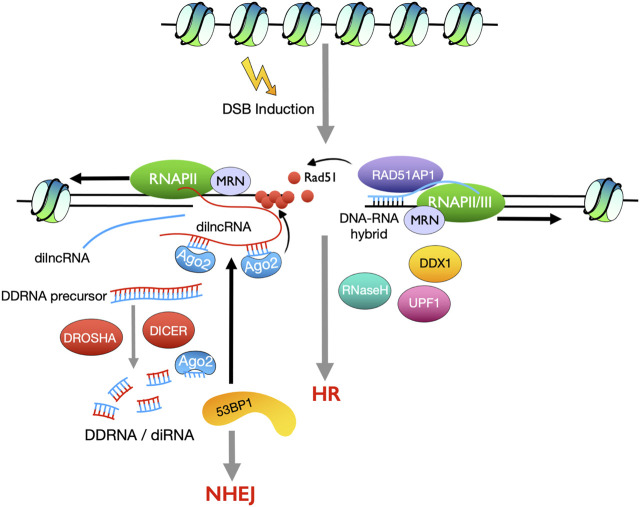
Summary of how non-coding RNAs induced by DSBs, promote DSB repair by NHEJ and HR. Small non-coding RNAs (diRNAs/DDRNAs) and long non-coding RNAs (dilncRNAs) generated from the vicinity of DSB sites promote 53BP1 recruitment and thus NHEJ repair (left). RNA-DNA hybrids, produced by RNAPII or RNAPIII around DSBs, promote DSB repair by HR.

A growing body of evidence shows that local DSB induced non-coding transcripts play important roles in HR (Durut and Mittelsten Scheid, 2019; Ohsawa et al., 2013). Small RNAs derived from the vicinity of DSBs, named DSB-induced RNAs (diRNAs) or DICER- and DROSHA-dependent small RNAs (DDRNAs), have been identified in both plants and vertebrates (Francia et al., 2012; Wei et al., 2012). DiRNAs promote HR repair in a manner dependent on the effector protein Argonaute 2 (Ago2) that is required for RAD51 recruitment (Gao et al., 2014). The production of diRNAs/DDRNAs indicates activation of non-coding transcription surrounding DSBs. Indeed, RNA polymerase II (RNAPII) is recruited to DSB sites, via its binding to the MRN complex, and generates damage-induced long non-coding RNAs (dilncRNAs). Most of the dilncRNAs are transcribed in the direction away from the DSB while less are transcribed towards the DSB to generate, which act not only as precursors to DDRNAs but also scaffolds for DDRNAs recruitment to the dilncRNA-from molecules (Michelini et al., 2017) (Figure 3). On the other hand, dilncRNAs and DDRNAs facilitate DDR focus formation and interact with 53BP1 (Michelini et al., 2017), suggesting a role of DDRNAs in promoting NHEJ repair (Figure 3).

### RNA-DNA Hybrids Promote Double-Strand Break Repair by Homologous Recombination

In fission yeast, RNAPII was shown to be rapidly recruited to a site-specific DSB induced by the endonuclease I-PpoI (Ohle et al., 2016). Remarkably, a robust increase of RNAPII around the DSB was detected, but augmentation of RNA transcripts was not observed by RT-qPCR at this region, which suggests the nascent transcripts immediately form RNA-DNA hybrids with the DNA template and are stabilized in these structures. Overexpression of RNaseH, *rnh1*, impairs DSB repair efficiency, indicating that the RNA-DNA hybrids act as a functional intermediate during HR repair (Ohle et al., 2016). Intriguingly, depletion of both RNaseH proteins, *rnh1* and *rnh201*, leads to the accumulation of RNA-DNA hybrids and inhibits HR-mediated DSB repair through impairing RPA recruitment to the ssDNA strand (Ohle et al., 2016). Therefore, the RNA-DNA hybrid intermediates must be tightly regulated, spatially and temporally, during HR repair. Furthermore, formation of RNA-DNA hybrids was observed at DSB sites in mammalian cells (Li et al., 2016). Consistently, depletion of the RNA-unwinding protein DEAD box 1 (DDX1) elevates the RNA-DNA hybrid levels and decreases RAD51 focus formation at DSBs, resulting in impaired DSB repair by HR (Li et al., 2016) (Figure 3).

It remains unclear how these RNA-DNA hybrids are formed and regulate DSB repair mediated by HR. Recently, RNA polymerase III (RNAPIII) was characterized as an essential factor in HR repair (Liu et al., 2021). RNAPIII is recruited to DSBs by the MRN complex and is required for the synthesis of the RNA strand, which protects the 3’ ssDNA overhang from degradation through the formation of RNA-DNA hybrids (Liu et al., 2021). Consistently, inhibition of RNA-DNA hybrid formation, by either knockdown of RNAPIII subunits or chemical inhibitors, significantly impairs end resection and focus formation of RPA and RAD51, which leads to defects in HR repair and thus loss of genetic material at DSBs (Liu et al., 2021). Interestingly, the formation of RNAPIII RNA-DNA hybrids requires the nuclease activity of CtIP and MRN and is restricted to S and G_2_/M phases (Liu et al., 2021), which is consistent with their roles in promoting HR repair. In this study, the nascent RNA strand produced by RNAPIII is proposed to be degraded for subsequent formation of ssDNA/RAD51 nucleoprotein filaments (Liu et al., 2021), whereas a role of RPA in this model needs further interrogation. Moreover, an *in vitro* system, in which mRNA synthesis occurs in the absence of promoters, shows that dilncRNAs are produced by RNAPII, but not RNAPIII, to form RNA-DNA hybrids at DSB sites (Sharma et al., 2021). Interestingly, DNA end melting mediated by MRN is required for dilncRNA synthesis, while the nucleolytic activity of MRN is dispensable for RNAPII transcription at DSBs (Sharma et al., 2021). At DSB sites induced by AsiSI, DROSHA promotes the production of RNA-DNA hybrids, probably by RNAPII, to facilitate DSB repair by HR or NHEJ (Castel et al., 2014; Lu et al., 2018). Paradoxically, no diRNAs/DDRNAs were detected using equal sequencing coverage and depth as seen in previous studies at DSB sites (Francia et al., 2012; Wei et al., 2012; Lu et al., 2018), which might be due to the different DSB induction systems that were utilized or the transient nature of these RNA species.

During HR repair, a ssDNA/Rad51 nucleoprotein filament invades the donor dsDNA homolog and forms a heteroduplex DNA joint called the D-loop (Petukhova et al., 2000; Van Komen et al., 2000). When the transcription machinery collides with the machinery mediating other physiological processes, such as DNA replication or DNA repair, the nascent RNA transcript forms hybrids with the template DNA and displaces the non-template ssDNA in the double helix, which generates a three-stranded structure called an R-loop (Rinaldi et al., 2020). Unscheduled R-loop formation is taken as a threat to genome stability, which has been thoroughly reviewed recently (Rinaldi et al., 2020). Some light has been shed on the mechanisms of how transcription enhances DNA end resection and thus HR repair via formation of D-loop and R-loop hybrids (DR-loops) (Ngo et al., 2021; Ouyang et al., 2021). In the study by Ouyang et al. (2021) the constitutive promoter of *sceGFP* in the direct-repeat GFP (DR-GFP) reporter was replaced with a tetracycline-inducible (Tet-On) promoter (Ouyang et al., 2021). When *sceGFP* was actively transcribed, HR efficiency was markedly enhanced although HR products were still detectable when transcription was inactive (Ouyang et al., 2021). In agreement, with Cas9-mediated DSB induction and transcriptional activation of a neuronal-specific gene, *ASCL1*, HR products were dramatically increased when transcription was active. Further, RNA transcripts were shown to stimulate HR by annealing with DNA mediated by the RAD51-associated protein RAD51AP1 (Ouyang et al., 2021). RAD51AP1 accumulates at DSB sites and promotes R-loop formation, which forms DR-loops together with D-loops generated by RAD51 strand invasion, to facilitate HR (Ouyang et al., 2021). At sub-telomeric DSB sites, the formation of R-loops and RNA-DNA hybrids, driven by an RNA/DNA helicase UPF1, stimulated end resection and thus HR (Ngo et al., 2021). R-loops and RNA-DNA hybrids are clearly generated *via* different mechanisms because the generation of R-loops is independent of DNA end resection but is induced at RNA-DNA hybrids that forms on ssDNA generated by resection (Ngo et al., 2021). In centromeres, RNA-DNA hybrids were also found to promote HR repair at DSBs in G_1_ phase (Yilmaz et al., 2021). These studies raise many interesting questions, for example how is the resolution of RNA-DNA hybrids regulated after DNA end resection? How are RPA and RNA-DNA hybrids coordinated to protect the 3’ ssDNAs and facilitate RAD51 deposition during HR repair? Do the RNA-DNA hybrids play a role in inhibiting NHEJ?

## Conclusion and Perspectives

In the last two decades, our knowledge of how epigenetic modifications regulate DSB repair, and DSB repair pathway choice has grown exponentially. Our knowledge of the interplay between the pro-NHEJ factor 53BP1 and pro-HR heterodimer BRCA1-BARD1 has increased extensively. The recruitment of 53BP1-RIF1-Shieldin to DSBs plays a critical role in promoting NHEJ repair. We now have an improved appreciation of how pre-existing and DSB-induced histone PTMs play pivotal roles in regulating DSB repair pathway choice in different phases of the cell cycle: Upon DSB induction, RNF168 is recruited to DSB sites to monoubiquitylate H2AK15, which is a prerequisite for both 53BP1 and BRCA1-BARD1 recruitment. In G_1_ phase, the pre-existing H4K20me1/2 on chromatin is recognized by 53BP1 to promote DSB repair by NHEJ. However, H4K20 is unmethylated on the post-replicative chromatin in S/G_2_ phase, which enhances the recruitment of BRCA1-BARD1 to facilitate HR repair. We have learned that in euchromatin and heterochromatin regions histone PTMs that promote transcriptional activation such as H3K36me and H3K4me2 and those that promote transcriptional repression such as H3K9me3, both promote DSB repair by HR through interactions with distinct factors. Intriguingly, we now appreciate that DSB-induced RNAs are involved in DSB repair regulation. The nascent RNA transcripts can also form R-loops with DNA ends at the breaks to promote HR repair.

It remains largely unknown how the intricate chromatin modification network, namely the chromatin landscape, is orchestrated to regulate DSB repair pathway choices. More studies are required to address whether other histone modifications, such as crotonylation and glycosylation, also contribute to DSB repair pathway choice. In addition, it is tempting to speculate whether DSB-induced small RNAs, diRNAs/DDRNAs, and dilincRNAs can stimulate heritable epigenetic changes on DNA molecules, such as DNA methylation, in the chromatin landscape surrounding the DSB sites, which may act as an “epigenetic memory” to prime the repaired region for more efficient repair if DSBs occur at the same sites. Indeed, DSBs do initiate the transient recruitment of the DNA methyltransferases DNMT1 and DNMT3B, in a SIRT1 dependent manner, and subsequent methylation at promoter regions (O'Hagan et al., 2008).

Why do the histone PTMs involved in DSB repair pathway choice, regardless of whether they occur in euchromatin or heterochromatin regions, tend to promote HR repair? Does this indicate that NHEJ repair is the default mechanism of DSB repair throughout the cell cycle and specific histone modifications are required to overcome the NHEJ default to promote HR? Indeed, NHEJ was found, by live imaging and fluorescent labeling of components in HR and NHEJ repair pathways, to be the dominant repair pathway not only in G_1_ but also in G_2_ phase where both HR and NHEJ repair pathways are functional (Karanam et al., 2012). Why then do cells choose a relatively more error-prone pathway as the main DSB repair mechanism? Compared to HR repair, NHEJ requires less processing at broken DNA ends such that it is more rapid and efficient, which helps to protect our genome from deleterious DSB-induced translocations or chromosome loss. Moreover, only ∼2% of our genome is protein-coding and the rest comprises mostly non-coding regions or regulatory elements such as introns, promoters, and enhancers. Even if nucleotide sequence changes are introduced at these non-coding regions by NHEJ, it is less likely to lead to mutation in genes or more detrimental consequences. On the other hand, it is interesting to envision why HR repair over NHEJ is used at centromeres in G_1_ phase without the presence of sister chromatids. Utilization of HR repair at repetitive sequences is thought to be detrimental to genome stability as it usually leads to repeat contraction and expansion (Khristich and Mirkin, 2020). However, Yilmaz et al. (2021) proposed activation of HR at centromeres in G_1_ counteracts the engagement of alternative mutagenic repair pathways, resulting in the prevention of chromosomal abnormalities (Yilmaz et al., 2021).

Interestingly, the highest occurrence of HR is at the peak of active replication with the longest half-lives of DSBs in mid S phase (Karanam et al., 2012), where sister chromatids are widely accessible due to the transient absence of histones on DNA. The proportion and rates of active HR vary widely at the DSBs among individual cells even at the same cell cycle phase (Karanam et al., 2012), which suggests that the choice between HR and NHEJ repair pathways could possibly be channeled by the unique chromatin landscapes surrounding the DSB sites in individual cells. Clearly the situation is very complex and hopefully future studies will provide a better understanding of the intriguing regulation of DSB repair processes.
